# Correlation of tumor‐infiltrating immune cells of melanoma with overall survival by immunogenomic analysis

**DOI:** 10.1002/cam4.3466

**Published:** 2020-09-15

**Authors:** Lili Huang, Hong Chen, Yu Xu, Jianhua Chen, Zhuqing Liu, Qing Xu

**Affiliations:** ^1^ Department of Oncology Shanghai Tenth People’s Hospital Tongji University School of Medicine Shanghai China; ^2^ Tongji University Cancer Center Shanghai China; ^3^ Department of Oncology, Dermatology Hospital Tongji University Shanghai China; ^4^ Department of Gastrointestinal Surgery Fujian Provincial Hospital Fuzhou China; ^5^ Department of musculoskeletal Oncology Fudan University Shanghai Cancer Center Shanghai China; ^6^ Department of Oncology Shanghai Medical College Fudan University Shanghai China

**Keywords:** CD8^+^ T cells, M2 macrophage, melanoma, tumor‐infiltrating immune cells

## Abstract

**Aims:**

Different types of tumor‐infiltrating immune cells not only augment but also dampen antitumor immunity in the microenvironment of melanoma. Therefore, it is critical to provide an overview of tumor‐infiltrating immune cells in melanoma and explore a novel strategy for immunotherapies.

**Methods:**

We analyzed the immune states of different stages in melanoma patients by the immune, stromal, and estimation of stromal and immune cells in malignant tumor tissues using expression data (ESTIMATE) scores. Immune cell types were identified by the estimating relative subsets of RNA transcripts (CIBERSORTx) algorithm in 471 melanoma and 324 healthy tissues. Moreover, we performed a gene set variation analysis (GSVA) to determine the differentially regulated pathways in the tumor microenvironment.

**Results:**

In melanoma cohorts, we found that ESTIMATE and immune scores were involved in survival or tumor clinical stage. Among the 22 immune cells, CD8^+^ T cells, M2 macrophages, and regulatory T cells (Tregs) showed significant differences using the CIBERSORTx algorithm. Furthermore, GSVA identified the immune cell‐related pathways; the primary immunodeficiency pathway, intestinal immune network for IgA, and TGF‐β pathways were identified as participants of the crosstalk in CD8^+^ T cells, Tregs, and M2 macrophages in the melanoma microenvironment.

**Conclusion:**

These results reveal the cellular and molecular characteristics of immune cells in melanoma, providing a method for selecting targets of immunotherapies and promoting the efficacy of therapies for the treatment of melanoma.

## Graphical abstract

Immunotherapy has shown excellent responses in melanoma, while the reaction is low. However, the molecular mechanisms of tumor infiltrated immune cells have not been explored. In this study, we found that higher ESTIMATE and immune scores were associated with a clinical‐stage in melanoma patients and also filtered the crosstalks between cells that immune cells. Our work revealed the immune cellular and molecular characteristics of melanoma, providing a method for selecting targets promoting immunotherapy efficacy.

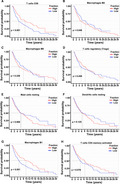

## 1 INTRODUCTION

Melanoma is relatively common worldwide and a life‐threatening form of skin cancer.^1^ The most effective therapy to treat melanoma is surgical resection.^2^ With the advances in immunology, immunotherapy has shown excellent outcomes in various diseases, especially melanoma.^3^ However, patient responses to nivolumab therapy have been reported to range from 10% to 20%.^4^ In most melanoma patients, clinical survival is reduced due to the metastases of advanced tumors.^5^ Therefore, it is crucial to understand the cellular and molecular characteristics of immune cells in melanoma and explore novel intervention targets as potential therapies, which might improve the efficacy of immunotherapy.

The tumor microenvironment (TME) consists of tumor cells, stromal tissues, vessels, and infiltrating cells, including T cells, B cells, and macrophages.^6^, ^7^ Different types of tumor‐infiltrating immune cells have been reported to not only augment but also dampen antitumor immunity in the melanoma microenvironment.^8^, ^9^ For example, CD8^+^ T cells, dendritic cells (DCs), and natural killer (NK) cells play essential roles in active immunity for antitumor therapies.^10^, ^11^, ^12^ Regulator T cells (Tregs) and tumor‐associated macrophages (TAMs) indicate immune suppression, which promotes tumorigeneis.^13^, ^14^ The use of traditional immunohistochemistry and flow cytometry for assessing tumor composition are limited due to their low throughput. Thus, a comprehensive evaluation of the tumor‐infiltrating immune cells in melanoma is crucial.

With the progress of high‐throughput sequencing technologies, bioinformatics techniques have been used to assess the composition of cells in different types of cancer.^15^ The estimation of stromal and immune cells in malignant tumor tissues using expression data (ESTIMATE) algorithm can predict immune states by calculating the immune and matrix scores of tumor tissues.^16^ Moreover, cell‐type identification by estimating relative subsets of RNA transcripts (CIBERSORT) can estimate the fraction of 22 different immune cells in tumor tissues based on the immune gene expression characteristics via bulk transcriptome profiles, and the CIBERSORTx, its extended version, was introduced in 2019 with a more accurate assessment via single‐cell experiments.^17^ These two deconvolution algorithms can provide an overview of the characteristics and mechanisms of tumor‐infiltrating immune cells in melanoma.

Immune cells exhibit different immune responses by direct and indirect cell‐cell contact mechanisms.^18^ In the B16 murine melanoma model, effector CD8^+^ T cells and activated NK cells cooperated and improved an immunotherapeutic response after treatment with interleukin‐2 (IL‐2) and cytotoxic T‐lymphocyte‐associated protein‐4 (CTLA‐4) blockade. Moreover, the depletion of M2 macrophages increased the surveillance of CD8^+^ T cells and promoted immunotherapy efficacy.^19^ However, the molecular mechanisms of the infiltrating immune cells and their correlations with clinical outcomes in TME have not been reported.

Here, we investigated the clinical survival of melanoma patients and the immune state of the melanoma tumor microenvironment using ESTIMATE algorithm analysis. We performed comprehensive subpopulations of tumor‐infiltrating immune cells and identified altered immune cells in two expression datasets using CIBERSORTx. Moreover, we used gene set variation analysis (GSVA) to explore the signaling pathways of immune cells in melanoma. Furthermore, we analyzed and selected immune cells and related pathways based on a multivariable logistic regression model, providing a novel strategy for filtering the crosstalk between cells. These results revealed the cellular and molecular characteristics of immune cells in melanoma and provide a method for selecting targets for immunotherapies, which may be crucial in improving the efficacies of therapies for the treatment of melanoma.

## 2 MATERIALS AND METHODS

### 2.1 Transcriptional expression profiles

The transcriptional data and corresponding clinicopathological and survival data from melanoma patients were downloaded from The Cancer Genome Atlas (TCGA) (https://cancergenome.nih.gov/). The dataset of normal skin with exposure to sunlight was collected from the Genotype‐Tissue Expression (GTEx) V7 release version (https://gtexportal.org/home/datasets) and TCGA and merged as a cohort. Complete information on healthy donors, including ages and genders, are described in the GTEx official annotation. The datasets contain 471 melanoma samples from melanoma patients and 324 skin samples from healthy donors. We then merged the data and removed the batch effect between GTEx and TCGA data using the function “Combat” in R package “sva”.^20^, ^21^ A principal component analysis (PCA) was performed and visualized before and after batch effect removal to ensure that the analysis was successful.

### 2.2 Immune and stromal scores

Immune scores, stromal scores, and tumor purity were calculated using the ESTIMATE algorithm.^22^ The clinicopathological parameters of melanoma patients, including the American Joint Committee on Cancer (AJCC) stages in 471 patients with skin cutaneous melanoma (SKCM) from TCGA (TCGA‐SKCM), were analyzed according to the immune, stromal, and ESTIMATE scores. The patients were divided into two groups according to the median values of immune, stromal, and ESTIMATE scores. Survival rates were analyzed in a high and low group of different immune, stromal, and ESTIMATE scores.

### 2.3 Tumor‐infiltrating immune cells

The CIBERSORTx algorithm was used to calculate and analyze the tumor‐infiltrating immune cells involved in melanoma and healthy donors. Twenty‐two immune cell subtypes were obtained and parsed from the annotated gene signature matrix LM22 and 100 permutations of the CIBERSORTx web portal (http://cibersortx.stanford.edu/). The 22 immune cells included T cells, B cells, NK cells, macrophages, dendritic cells, mast cells, eosinophils, and neutrophils. All samples were counted and analyzed using the CIBERSORTx *P*‐value and root mean square error (RMSE). Only cases with *P* < .05 were filtered and selected for the subsequent analysis. Immune cell fractions from TCGA and GTEx were filtered and analyzed using the CIBERSORTx algorithm. The Wilcoxon test was used to examine the differences between the immune cell fractions from melanoma and healthy control samples.

### 2.4 Correlation of tumor‐infiltrating immune cells in melanoma tissues

Immune cell files from TCGA‐SKCM were selected for clinical analysis. The data on filtered immune cells were merged with the survival rate, and a correlation curve was drawn for the pathophysiology of melanoma. Correlations among purified immune cells were also evaluated and implied.

### 2.5 Immunofluorescence analysis of immune cells

To confirm the results of genomic analysis, we performed immunofluorescence (IF) staining. The expression levels of immune cells in both melanoma and healthy tissues were recorded and analyzed. Slides with 5‐mm sections were obtained from samples, fixed with paraformaldehyde, and embedded in paraffin. Antigen retrieval was performed using a heated antigen unmasking solution. The primary antigens were incubated overnight at 4°C after 1 hour serum blocking, and secondary antibodies (Alexa Fluor 555 (Cell Signaling Technology, USA)) were incubated for 1 hour at room temperature. The antibodies used in these experiments were CD8, CD206, CD68, and Foxp3. DNA was stained with ProLong^TM^ gold antifade mountant with DAPI (ThermoFisher, USA) for 5 minutes in the dark. Images were determined using a Leica fluorescence microscope (Leica Microsystems, Germany).

### 2.6 Immune‐related pathway analysis of TCGA cohorts

We performed GSVA to reveal the underlying changes in signaling mechanisms using R package.^23^ The differentially expressed KEGG pathways were identified from the data of melanoma and healthy tissues in TCGA‐SKCM and GTEx cohorts, respectively. The gene set c2.cp.kegg.v6.2.symbols.gmt was downloaded from Molecular Signatures Database (MSigDB) and set as the reference gene list.^24^ After inputting the gene expression profile matrix, the GSVA algorithm transformed the genes of the matrix into scores that represented each KEGG pathway's activity based on the reference gene. Then, the differentially activated pathways between TCGA‐SKCM and GTEx cohorts were determined by R package limma with log_2_ fold change (FC)> 2 or < −2 and *P*‐value < .05. After comparing the GSVA scores of melanoma and healthy donors.^25^ The correlations of immune‐related genes and KEGG pathways were filtered and analyzed by Spearman correlation analysis.

### 2.7 Statistical analysis

All statistical analyses were performed using R software and GraphPad Prism v.6.0. The ESTIMATE algorithm was used to calculate the immune and stromal scores in melanoma. Kaplan‐Meier curve analysis and log‐rank tests were performed to observe the survival rate of melanoma patients. The R package corrplot was used to display the correlations among different immune cells. The Wilcoxon test was used to identify the relationship between filtered immune cells and KEGG pathways. Data points are shown as the mean ± standard error of the mean (SEM) of biological replicates. Statistical significances are indicated as: * *P* < .05, ** *P* < .01, *** *P* < .001 or **** *P* < .0001. Two‐tailed paired or unpaired Student's *t*‐tests and one‐ or two‐way ANOVA with multiple comparisons were performed to determine significant differences in normally distributed data.

## 3 RESULTS

### 3.1 Immune and stromal scores at different stages of melanoma

**Figure 1:**
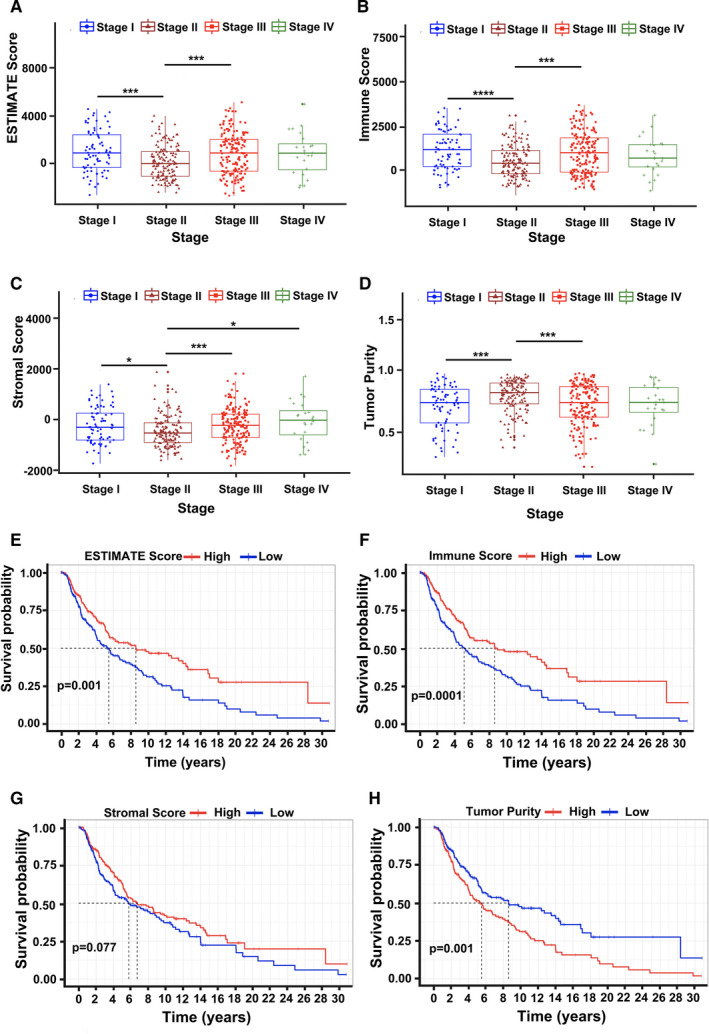
ESTIMATE, stroma, and immune scores in TCGA‐SKCM of melanoma patients by ESTIMATE algorithm analysis. (A–D): ESTIMATE, stroma, and immune scores and tumor purity, respectively, at different stages. The lowest ESTIMATE, stroma, and immune scores were in stage II. (E–H): Association between survival curves and different ESTIMATE, stroma, and immune scores and tumor purity, respectively, in melanoma patients in TCGA‐SKCM. (n = 471, * *P* < .05, *** *P* < .001, and **** *P* < .0001)

Datasets of 471 tumor samples from TCGA‐SKCM and 324 skin samples from GTEx were downloaded and merged for further analysis. Batch effects were removed, and the plots generated using PCA before and after the removal are shown in Figure S1. ESTIMATE, immune, and stromal scores were calculated using the ESTIMATE algorithm and ranged from −2000 to 5000 (Figure 1A–C). The lowest ESTIMATE scores were found in stage II; this was the same in immune and stromal scores (Figure 1A–C). ESTIMATE scores were significantly associated with stage III but not with stage IV (Figure 1A). The same trend was observed in the immune score of melanoma patients (Figure 1B). Stromal scores indicated that patients in the late stage III, stage IV, or very early stage (I) of melanoma had higher stromal scores than those in stage II (Figure 1C). The tumor purity of melanoma tissues presented a high association with clinical stage II (Figure 1D); this was opposite to the immune scores. These results showed that higher tumor purity corroborated with a lower immune score in melanomas (Figure 1B–D).

To explore the potential role of the ESTIMATE, stromal, and immune scores in overall survival (OS), we divided and analyzed the dataset of 471 patients from TCGA‐SKCM into high and low score groups. In Figure 1E, higher ESTIMATE scores were significantly associated with OS. Interestingly, the survival curves showed that a higher immune score indicated a better prognostic implication than that in the low immune score group (Figure 1F). The results were similar to the immune score at different clinical stages (Figure 1B). While survival curves indicated no association between different stromal score groups in melanoma (Figure 1G), tumor purity curves indicated that high tumor purity was significantly associated with poor survival (Figure 1H). These results show a significant association between the immune microenvironment and melanoma stage or overall survival.

### 3.2 Composition of immune cells in melanoma and normal tissues

**Figure 2:**
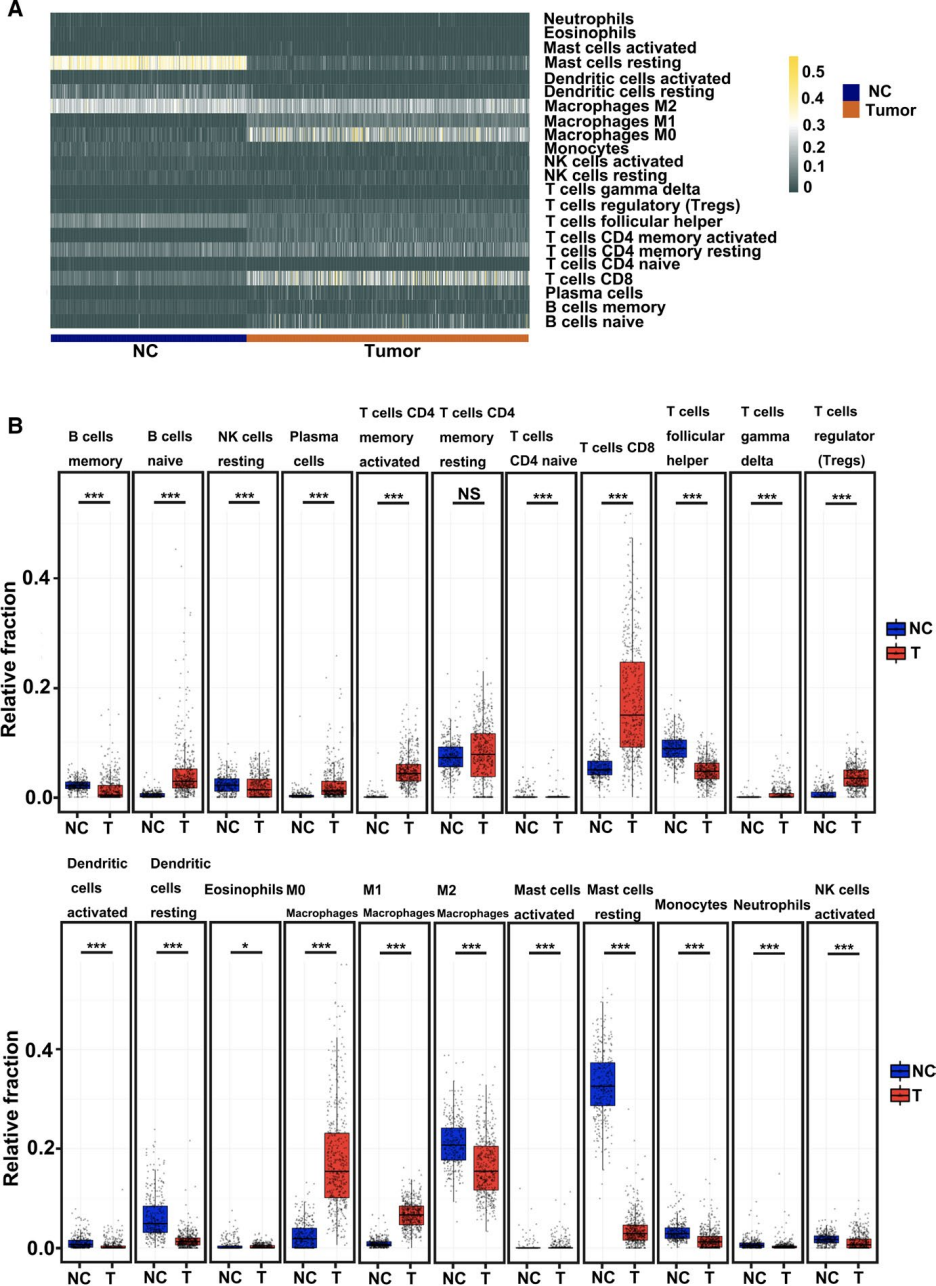
Analyses of immune cells between melanoma and healthy tissues using the CIBERSORTx algorithm. (A) Heatmap of normalized absolute abundance of immune cell types in an individual sample. (B) Boxplots of different immune cells between melanoma and healthy control samples. The blue boxplot (NC) represents healthy control; the red boxplot (T) represents tumors. (* *P* < .05    and *** *P* < .001)

To investigate the composition of infiltrating immune cells, we used CIBERSORTx. An overview of the infiltrated immune cells is shown in Figure 2. After using the CIBERSORTx algorithm, the distribution of immune cells was demonstrated in a heatmap, revealing different fractions in immune cells (Figure 2A). The bar plot results indicated a high proportion of resting mast cells, resting dendritic cells, and M2 macrophages in healthy skin tissues (Figure S2). Plasma cells, B cells, CD8^+^ T cells, M0 macrophages, and Tregs accounted for high fractions in melanoma tissues (Figure S2). Gene clustering indicated that resting mast cells, resting dendritic cells, B‐cell memory, and M2 macrophages were decreased in melanoma tissues (Figure S2). However, in the adaptive immune system of melanoma, increased proportions of CD8^+^ T cells, M0 macrophages, M1 macrophages, and Tregs were observed (Figure 2A). As shown in Figure 2B, resting memory CD4^+^ T cells showed no significant difference between tumors and healthy tissues. The other 21 types of immune cells, including CD8^+^ T cells, Treg cells, M0 macrophages, M1 macrophages, and M2 macrophages were significantly enriched in melanoma tissues than in healthy tissues. Moreover, CD8^+^ T cells, M0 macrophages, and M2 macrophages accounted for the top three types among the 22 types of tumor‐infiltrating immune cells (Figure 2B).

**Figure 3:**
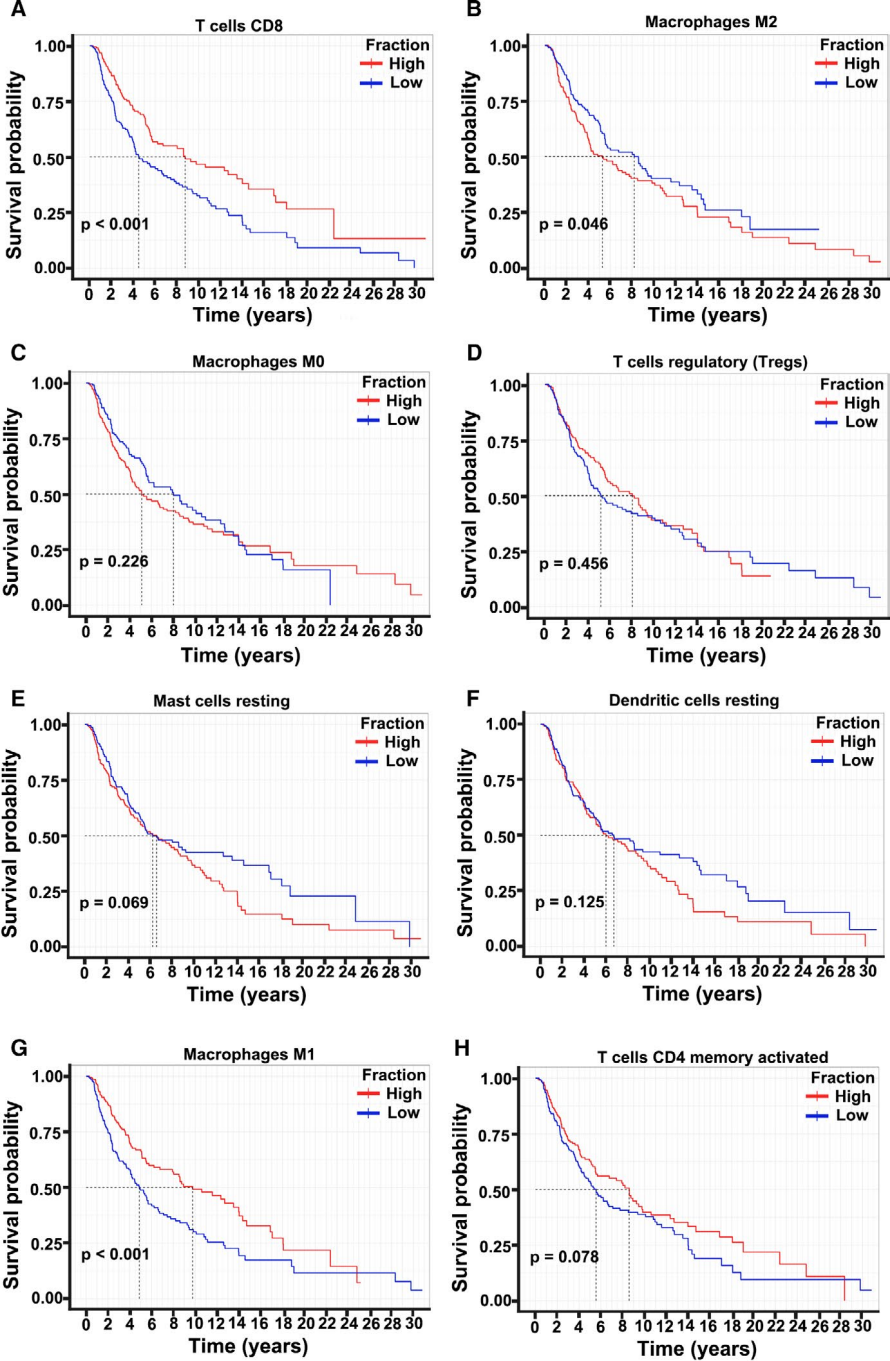
Association between survival curves and the different fractions of immune cells, including CD8^+^ T cells (A), M2 macrophages (B), M0 macrophages (C), and Tregs (D) in melanoma patients in TCGA‐SKCM. Survival curves of resting mast cells (E), resting dendritic cells (F), M1 macrophages (G), and activated memory CD4^+^ T cells (H) in melanoma patients in TCGA‐SKCM. The number of tumor patients and healthy control participants was 471 and 324, respectively.‐

We also used Kaplan‐Meier survival analysis to identify the association of tumor‐infiltrating immune cells with survival outcomes (Figure 3). Results showed that patients with a high proportion of tumor‐infiltrating CD8^+^ T cells had better overall survival than those with a low percentage of tumor‐infiltrating CD8^+^ T cells in the TCGA‐SKCM datasets (Figure 3A). However, survival curves revealed that a high proportion of M2 macrophages predicted poor overall survival in melanoma tissues (Figure 3B). No significant differences in the OS between melanoma tissues and infiltrating M0 macrophages (Figure 3C) were observed. Moreover, we established the survival curves of Tregs in melanoma, which thereby indicated that Tregs play a crucial role in tumor progression (Figure 3D). Surprisingly, Tregs presented no significant OS in patients with melanoma (Figure 3D). Furthermore, survival analysis was also established for other immune cells, including resting mast cells, resting dendritic cells, M1 macrophages, and memory CD4^+^ T cells (Figure 3E‐H). Resting mast cells, resting dendritic cells, and memory CD4^+^ T cells showed no significant OS in melanoma patients (Figure 3E–H). However, a high proportion of M1 macrophages presented excellent overall survival in melanoma tissues. M1 macrophages were opposite to those of M2 macrophages. These results suggest that tumor‐infiltrating CD8^+^ T cells, M2 macrophages, and M1 macrophages play essential roles in tumor progression.

### 3.3 CD8^+^ T cells and macrophages in melanoma tissues

**Figure 4:**
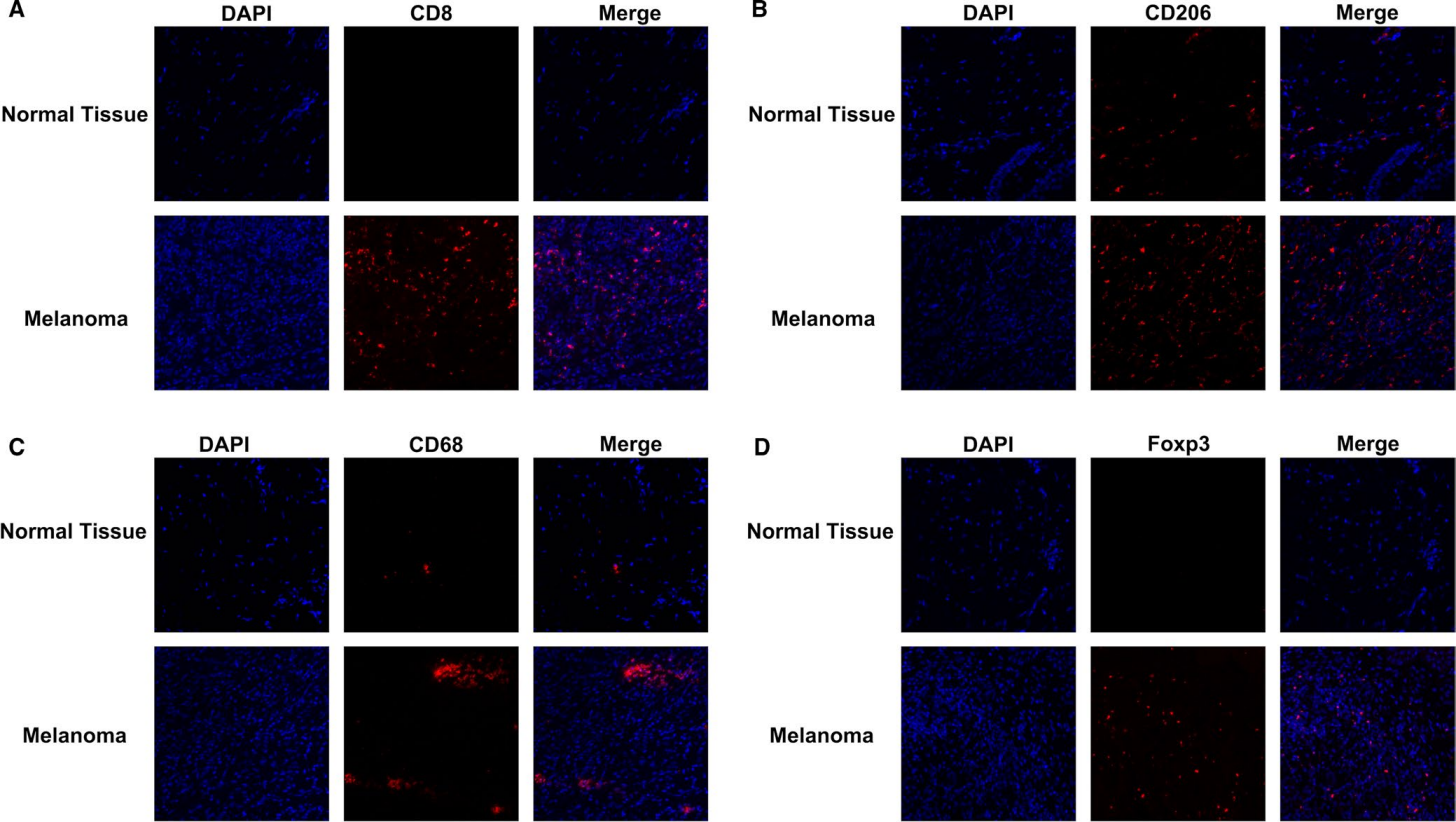
Assessment of immune cells in melanoma. Immunofluorescence analysis of CD8, CD206, CD68, and Foxp3 expression in melanoma and normal skin tissue samples. Representative images from three patients displayed positive staining in the stromal regions. CD8 (A), CD206 (B), CD68 (C), and Foxp3 (D) staining. × 20X magnification

To confirm the CIBERSORTx algorithm analysis results, we performed Immunofluorescence staining on M0 macrophages, M2 macrophages, CD8^+^ T cells, and Tregs in melanoma tissues (Figure 4). The IF staining showed that healthy skin was characterized by low immune infiltration, whereas tumor tissues recruited more immune cells, including CD8^+^ T cells, Tregs, M0 macrophages, and M2 macrophages (Figure 4). Higher CD8‐positive cells were identified in melanoma tissues than those in healthy tissues (Figure 4A). We used CD206 as an M2 macrophage marker to evaluate the expression of tumor‐associated macrophages in melanoma. Surprisingly, the healthy skin tissues were characterized by a low‐positive expression of the immune marker CD206, whereas melanoma tissues exhibited a high expression of CD206 (Figure 4B). These results were in contrast with the downregulation trend of M2 macrophages in the CIBERSORTx algorithm analysis. Moreover, other immune cells presented higher CD68 (M0 macrophages), and Foxp3 (Tregs) expression in melanoma tissues than in healthy skin tissues (Figure 4C–D). The IF staining analysis confirmed the significant increase in M0 macrophages and Tregs in melanoma (Figure 4C–D). CIBERSORTx algorithm analysis revealed that immune cells were continuously recruited into the tumor microenvironment during the development of melanoma.

### 3.4 Co‐expression of tumor‐infiltrating immune cells

**Figure 5:**
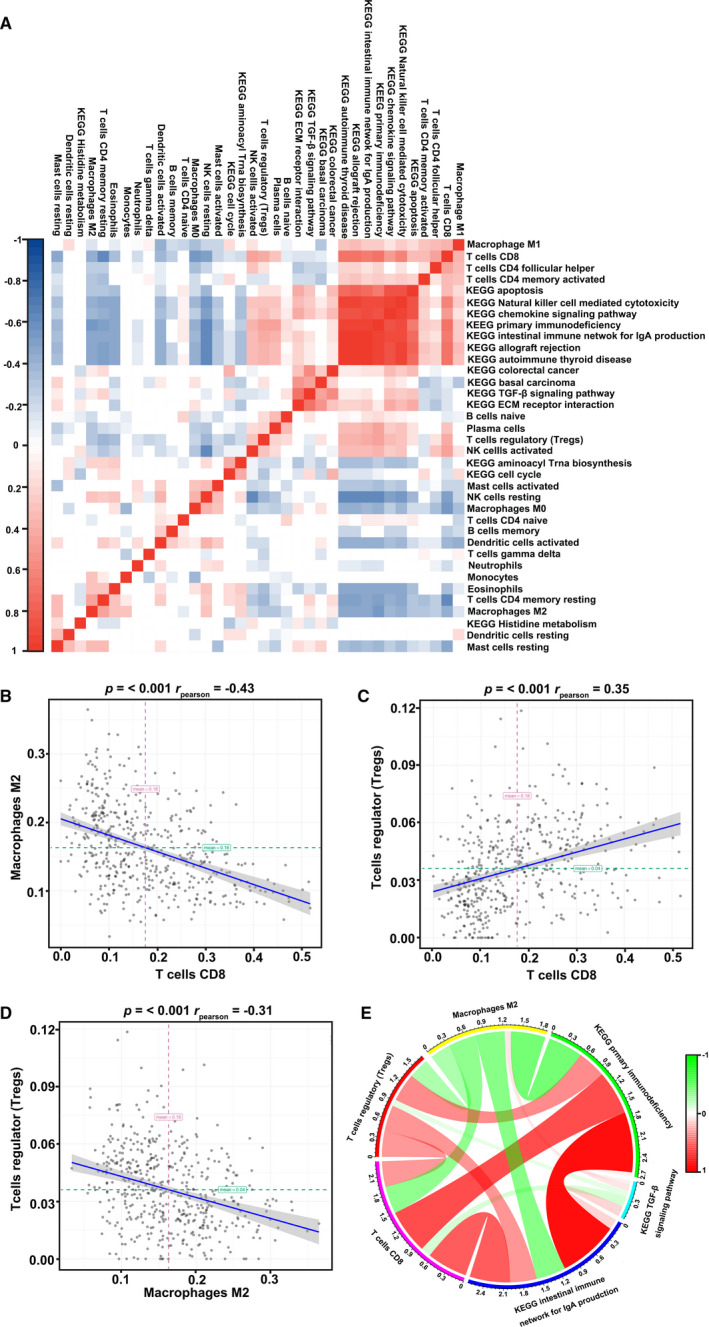
Co‐expression of immune cells in melanoma tissues. (A) Correlation heatmaps of immune cells and altered pathways in melanoma tissues. Relationship between CD8^+^ T cells and M2 macrophages (B), CD8^+^ T cells and Treg (C), and M2 macrophages with Treg (D) in melanoma tissues after analysis. (E) Circos plot of the relationship between altered immune cells CD8^+^ T cells, M2 macrophages, Tregs, and the intestinal immune network for IgA, primary immunodeficiency, and TGF‐β signaling pathway

To evaluate the co‐expression among 22 different types of tumor‐infiltrating immune cells, we used Spearman correlation to analyze the melanoma samples’ immune profiles in TCGA‐SKCM cohorts (Figure 5A). The results indicated that 22 different cells presented weak or strong correlations in the tumor tissues of TCGA‐SKCM cohorts. B cells, mast cells, neutrophils, and monocytes showed a weak relationship with other immune cells in the TCGA‐SKCM cohort (Figure 5A); whereas other immune cells such as CD8^+^ T cells and M0 presented a strong relationship. To explore the potential interaction network, we identified the cells with a correlation coefficient > 0.3 in the melanoma‐infiltrating immune cells. Using the combined above results, we selected CD8^+^ T cells, M2 macrophages, and Tregs for further investigation. The three immune cell types with the highest positive correlations with CD8^+^ T cells were M1 macrophages, Tregs, and activated NK cells, whereas M0 macrophages, resting memory CD4 T cells, and M2 macrophages showed a negative correlation with CD8^+^ T cells (Figure 5A). Tregs were positively associated with CD8^+^ T cells in the TCGA‐SKCM cohorts, whereas the correlation with M2 macrophages was negative (Figure 5A). Moreover, M2 macrophages showed an inverse relationship with other immune cells with reference to CD8^+^ T cells in the TCGA‐SKCM cohorts. The correlation curve indicated that M2 macrophages were significantly associated with CD8^+^ T cells, thereby indicating a negative interaction in melanoma (Figure 5B; *P* < .001, r_pearson_ = −0.43). Tregs and CD8^+^ T cells’ correlation revealed that these two immune cells have a close interaction in melanoma datasets (Figure 5C). M2 macrophages showed a strong negative correlation with Tregs in the TCGA‐SKCM samples (Figure 5D; *P* < .001, r_pearson_ = −0.31). Therefore, these data imply that M2 macrophages, CD8^+^ T cells, and Tregs are linked in the melanoma tumor microenvironment.

### 3.5 KEGG pathway analysis in melanoma tissues

KEGG pathway analysis was performed to explore the potential mechanism of the tumor‐infiltrating immune cells in melanoma using GSVA. GSVA results showed that 48 pathways were highly enriched in the melanoma group (Figure S3). On the other hand, 52 routes were found to be downregulated in melanoma cohorts compared to those in healthy skin tissues (Figure S3). Among these differential pathways, we focused on 14 KEGG pathways related to immune mechanism, including 10 upregulated pathways and four downregulated pathways in melanoma (Figure S3). Pathways and immune cells overlapped in GSVA for co‐expression analysis (Figure 5A). CD8^+^ T cells presented a negative correlation with five signaling pathways and showed a positive interaction with eight pathways, including primary immunodeficiency pathways (Figure 5A). The routes with negative correlation with Tregs are cell cycle, aminoacyl tRNA biosynthesis, and TGF‐β signaling. Similarly, Tregs showed the same positive trends with 8 KEGG pathways as CD8^+^ T cells in the TCGA‐SKCM datasets (Figure 5A). However, the associations of Tregs with these pathways were weak compared to those of CD8^+^ T cells (Figure 5A). Furthermore, M2 macrophages presented a positive correlation with pathways related to the TGF‐β signaling pathway, aminoacyl tRNA biosynthesis pathway, and colorectal cancer in melanoma cohorts (Figure 5A). We identified seven routes with a positive correlation with M2 macrophages. Therefore, we infer that CD8^+^ T cells, Tregs, and M2 macrophages might influence the crosstalk in melanoma progression.

### 3.6 Correlations of KEGG pathways and tumor‐infiltrating immune cells

**Figure 6:**
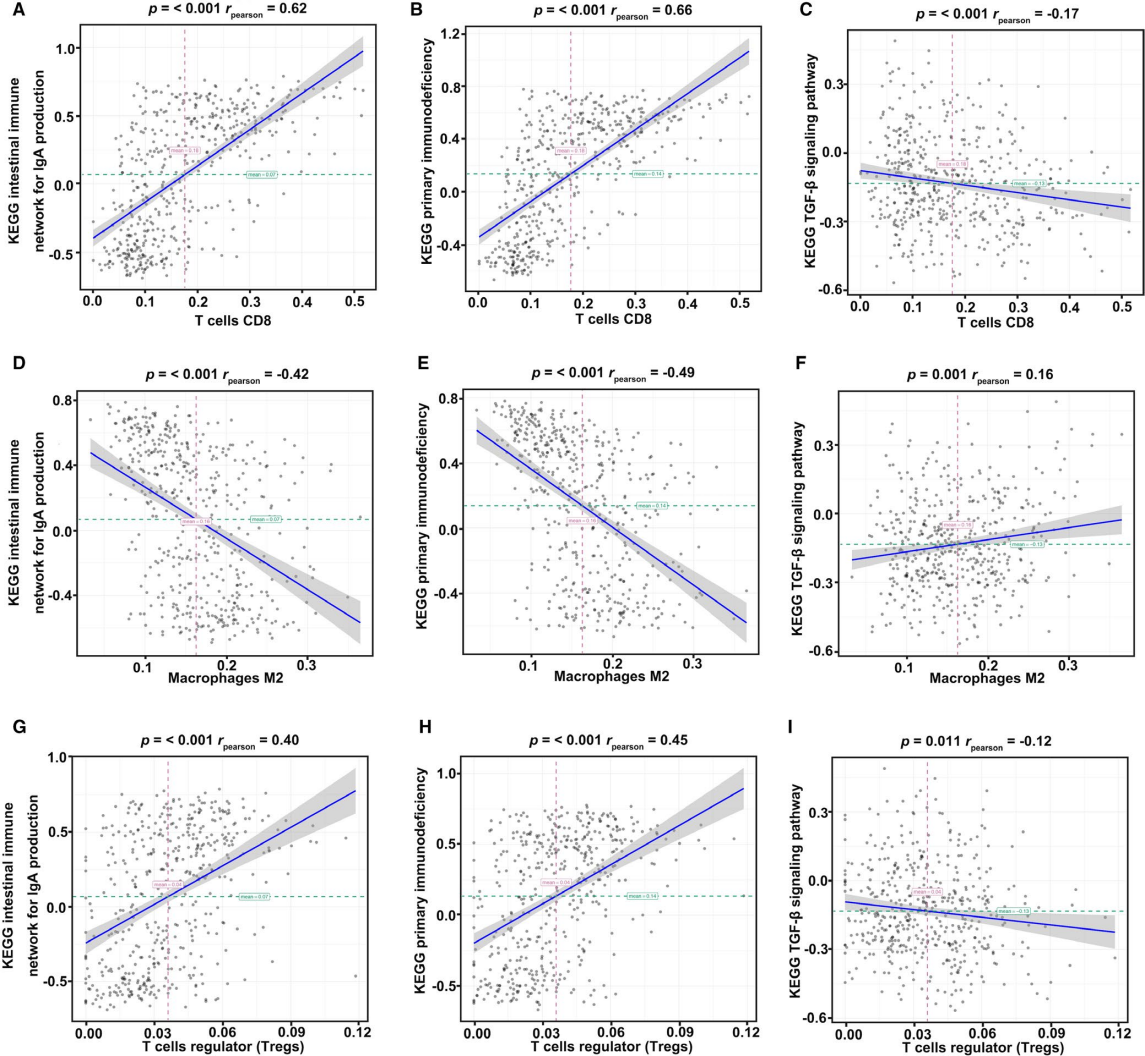
Correlation between immune cells and pathways in melanoma. CD8^+^ T cells (A–C) and Tregs (G–I) showed a positive correlation with the intestinal immune network for IgA, primary immunodeficiency pathways, and a negative correlation with the TGF‐β signaling pathway. M2 macrophages (D–F) showed a negative correlation with CD8^+^ T cells and Tregs

To comprehensively analyze the mechanism of immune cells in the tumor immune microenvironment, we selected three pathways for further exploration using GSVA (Figure 5E). These immune‐associated pathways include the intestinal immune network for IgA, primary immunodeficiency, and the TGF‐β signaling pathway (Figure 5E). The list of genes contributing to the core enrichment of the three selected pathways is shown in Table S1. CD8^+^ T cells showed a high correlation with the intestinal immune network for IgA and primary immunodeficiency and a weak relationship with the TGF‐β signaling pathway (Figure 5E). Similarly, the degrees of interaction between M2 macrophages and pathways were the same as those of CD8^+^ T cells. As described earlier, immune cells with a correlation coefficient > 0.3 indicate a high degree of relationship with melanoma (Figure 6). CD8^+^ T cells, M2 macrophages, and Tregs had an absolute value of correlation > 0.4 with the intestinal immune network for IgA and primary immunodeficiency (Figure 6A–H). However, TGF‐β pathways showed weak correlations with CD8^+^ T cells, M2 macrophages, and Tregs in melanoma (Figure 6C–I). CD8^+^ T cells presented a strong connection with the intestinal immune network for IgA and primary immunodeficiency; this was consistent with the role of CD8^+^ T cells in the TME (Figure 6A–B). The M2 macrophage data showed a strong negative correlation, implying that M2 macrophages have an inverse relationship in natural kill efficacy (Figure 6D–E). These results indicate that CD8^+^ T cells, Tregs, and M2 macrophages participate in the crosstalk of the intestinal immune network, especially those of IgA or primary immunodeficiency. As TGF‐β plays an essential role in the development and progression of tumor environment, we analyzed the regulatory role of immune cells in the TGF‐β pathway (Figure 6C–I). Consistent with previous studies, a negative correlation between CD8^+^ T cells and the TGF‐β pathway was observed; this indicates that CD8^+^ T cells play a crucial role in cytotoxicity function (Figure 6C). However, M2 macrophages were weakly positive for the TGF‐β pathway, promoting tumor progression (Figure 6F). Thus, we hypothesized that M2 macrophages induces tumor progression and inhibits the regulation of CD8^+^ T cells through the TGF‐β pathway. Further studies are needed to identify the crosstalk of the TGF‐β pathway with CD8^+^ T cells and M2 macrophages. Altogether, these data reveal that CD8^+^ T cells, Tregs, and M2 macrophages can interact with one another in the melanoma microenvironment through the primary immunodeficiency pathway, the intestinal immune network for IgA, or the TGF‐β pathway.

## 4 DISCUSSION

Tumor‐infiltrating immune cells not only augment but also dampen antitumor immunity through immune response or cytokine release in the tumor microenvironment.^8^, ^9^ Thus, it is critical to provide an overview of tumor‐infiltrating immune cells in melanoma and explore a novel strategy for immunotherapy. In this study, we investigated the clinical survival of patients and the immune state of the melanoma tumor microenvironment using ESTIMATE algorithm analysis. A higher ESTIMATE and immune scores were associated with a clinical stage in melanoma patients. We performed comprehensive subpopulations of tumor‐infiltrating immune cells and identified altered immune cells in two datasets using CIBERSORTx. TCGA‐SKCM datasets and IF staining confirmed that CD8^+^ T cells, M0 macrophages, M2 macrophages, and Tregs differently infiltrated into melanoma tissues. We analyzed and selected immune cells and related pathways based on a multivariable logistic regression model, thereby providing a novel strategy for filtering the crosstalk between cells. The results of GSVA demonstrated that CD8^+^ T cells, Tregs, and M2 macrophages could interact with one another in the TME through the primary immunodeficiency pathway, the intestinal immune network for IgA, or the TGF‐β pathway. These results revealed the cellular and molecular characteristics of immune cells in melanoma, providing a method for selecting targets of immunotherapies and promoting the efficacy of therapies for the treatment of melanoma.

The ESTIMATE score is a method for evaluating the different types of cells in the tumor microenvironment, which is significantly associated with the biological characteristics of tumors.^22^ The ESTIMATE algorithm analysis has been used to estimate stromal cells and tumor‐infiltrating immune cells in different cancers using gene expression data with calculated stromal and immune scores.^26^ In this study, we combined the ESTIMATE, stromal, and immune scores at varying stages of melanoma in patients. Our results indicate that stromal and immune scores are associated with clinical features in the tumor microenvironment. Interestingly, we also found that stage II patients received the lowest scores in the ESTIMATE, stromal, and immune scores analysis. In the tumor microenvironment, tumor‐infiltrating cells interact with tumor cells, which promote tumor progression and metastasis by regulating the balance between tumor cells and immune cells.^14^ Primary stage I and II melanoma patients are associated with chronic inflammatory cells, including numerous tumor‐infiltrating lymphocytes.^27^ Moreover, several studies have reported that lymphocytes must be infiltrated and disrupted in the vertical growth phase as tumor cells grow.^28^ While patients with stage II melanoma may experience tumor recurrence after treatment, tumor cells grow faster than tumor‐infiltrating immune cells.^29^ For stage III or IV cutaneous melanoma with lymph node metastases, tumor‐infiltrating immune cells are particularly complex.^30^ Lymph nodes are rich in CD3 and CD20 lymphocytes, making the definition of immune score much more difficult.^31^ Indeed, the scoring for the lymph node metastases in patients requires a new algorithm for normalization against different clinical stages. Different proportions and types of tumor‐infiltrating cells were largely altered in various melanoma stages, resulting in stromal and immune scores. Furthermore, we also found that higher ESTIMATE and immune scores corroborated with longer OS in melanoma patients. These results indicate that the TME has different immune states at different disease stages, and that these different immune states correlate with survival rate. Therefore, a comprehensive evaluation and classification of immune cells in the TME in melanoma tissue might be crucial for guiding immunotherapy.

To understand the immune state and classify the types and functions of immune cells in the TME, we used the CIBERSORTx algorithm to explore the immune cells in melanoma. Our results identified that CD8^+^ T cells, M0 macrophages, M2 macrophages, and Tregs are differentially expressed in melanoma among the 22 types of tumor‐infiltrating immune cells. The IF staining of melanoma samples confirmed the differences among CD8^+^ T cells, M0 macrophages, M2 macrophages, and Tregs between tumor tissues and healthy tissues. Tumor‐infiltrating immune cells are associated with tumor suppression, immunosuppression, and tumor‐associated proliferation and metastasis in therapy.^10^, ^11^, ^12^ At the early stage of the disease, CD8^+^ T cells differentiate into cytotoxic T lymphocytes (CTLs) and combine with NK cells to exert an efficient antitumor response by directly targeting tumor cells.^32^ However, activated NK cells are present in higher proportions in healthy tissues than in melanoma tissues. NK cells are innate lymphocytes that are activated in the early immune response of various diseases.^33^ Recent studies have identified that NK cells are activated and expanded in skins during infections.^34^ Moreover, metabolism can reprogram the function and phenotypes of NK cells in the TME.^35^ Therefore, mechanisms in the early response of NK cells in the human skin or melanoma should be explored. Tumor‐infiltrating Tregs prevented antitumor immunity and promoted tumor progression via various mechanisms of immunosuppression in TME.^36^ High levels of tumor‐associated M2 macrophages are significantly associated with reduced survival rates.^37^ Our results indicate that CD8^+^ T cells, M0 macrophages, and Tregs are significantly expressed in the tumor tissue and might play essential roles in melanoma progression. Surprisingly, tumor‐associated M2 macrophages presented higher CD206 expression in melanoma tissues than in healthy tissues. Moreover, the CIBERSORTx algorithm analysis indicated a decrease in the proportion of M2 macrophages in melanoma. Similar to melanoma cells, M2 macrophages and Tregs cooperate to form an immunosuppressive microenvironment in melanoma.^38^ A study has suggested that M2 macrophages could be divided into regulatory and wound‐healing macrophages in different tissues.^39^ M2 subtypes were described as M2a, M2b, and M2c subtypes which can be activated by classical or alternative macrophage activation.^40^ The results of the genomic datasets did not reflect all the subtypes of M2 macrophages. Hence, how these immune cells work and interact with one another remains unknown.

Tumor‐infiltrating immune cells exert different immunosuppression by direct and indirect cell‐cell contact mechanisms.^13^, ^14^ Correlations among the 22 different tumor‐infiltrating cells indicated that Tregs had a positive association with CD8^+^ T cells in the TCGA‐SKCM cohorts.^10^ Tregs can suppress nonessential immune responses such as those seen during transplantation.^41^ Tregs prevent an effective immune response and thus present an immunosuppressive effect.^42^ We determined that Tregs had a strong positive correlation with CD8^+^ T cells; this occurrence might be consistent with tumor states. Moreover, Tregs have been reported to suppress in the interferon‐γ secretion of CD8^+^ T cells; this reduces the efficacy of antitumor immunotherapy.^42^ Thus, it is vital to explore the mechanism between CD8^+^ T cells and Tregs to promote immunotherapy efficacy in treating melanoma. Studies have shown that a reduction in macrophages improved CD8^+^ T cell surveillance and increased sensitivity to anti‐PD‐1 treatment.^19^ Our correlation analysis of the TCGA‐SKCM showed that M2 macrophages have a strong negative correlation with CD8^+^ T cells in melanoma. These findings were similar to those of previous studies, in which the interaction of M2 macrophages and CD8^+^ T cells was implied in the melanoma tumor microenvironment during tumor progression. KEGG pathway analysis showed that M2 macrophages and CD8^+^ T cells had a strong correlation with some pathways, such as the TGF‐β pathway. The activation of the interferon pathway may activate CD8^+^ T cells and reduce TAMs in the TME, which would thereby increase the efficacy of immunotherapy.^43^ Thus, understanding the crosstalk between CD8^+^ T cells and M2 macrophages is essential for guiding immunotherapy. Our results identified three pathways among CD8^+^ T cells, M2 macrophages, and Tregs in the melanoma microenvironment. IL‐6 is one of the core enrichment genes of the intestinal immune network for the IgA pathway. IL‐6 and TGF‐β have been identified as the primary activators of *MAF* on CD8^+^ T cells, contributing to the suppression of melanoma microenvironment.^44^ Moreover, IL‐6 induced CD4^+^Foxp3^+^ Treg migration into tumor sites by upregulating CXCR1 expression.^45^ Studies have also shown that tumor‐derived IL‐6 promotes the polarization of M2 macrophages in melanoma.^46^ However, the mechanism of IL‐6‐mediated the interaction among CD8^+^ T cells, Tregs, and M2 macrophages needs further studies. Similarly, the stimulatory molecule CD40 was identified in the primary immunodeficiency pathway to induce the exhaustion of CD8^+^ T cells,^47^ abrogate the expansion of Treg cells,^48^ and activate M2 macrophage proliferation.^49^ Furthermore, TGF‐β1 mediates M2 macrophage polarization as a core gene in the TGF‐β signaling pathway.^50^ Treg cells can produce the immunosuppressive cytokine TGF‐β1, promote self‐proliferation, and attenuate the effector function of human CD8^+^ T cells in the TME.^10^, ^51^ These strategies provide effective channels in which the potential mechanisms of tumor‐infiltrating immune cells in the progression of melanoma can be explored. Furthermore, GSVA analysis between tumor‐infiltrating immune cells and KEGGs indicated potential targets for immunotherapies, thereby promoting the efficacy of therapies in treating melanoma. Further work is needed to explore and confirm the regulation of altered pathways in different immune cells in melanoma. Above all, this study reveals the cellular and molecular characteristics of immune cells in melanoma, providing a more specific method for selecting targets of immunotherapies.

## CONFLICTS OF INTEREST

The authors declare no competing interests.

## AUTHOR’S CONTRIBUTION

ZL and QX designed the study. LH and HC developed the methodology. LH, YX, and JC collected data. LH and HC analyzed the data. LH wrote the paper and all other authors reviewed the paper.

## Supporting information

Fig S1‐S3

Table S1

## Data Availability

The data support findings of this study are available from the corresponding author upon request.
