# Supplementary material for: Correlation of tumor‐infiltrating immune cells of melanoma with overall survival by immunogenomic analysis
Source: Cancer Med. 2020 Sep 15;9(22):8444–56. doi: 10.1002/cam4.3466 (PMC7666744; doi:10.1002/cam4.3466)
Supplement: Supplementary file 1 — Fig S1‐S3 [file CAM4-9-8444-s001.pdf]

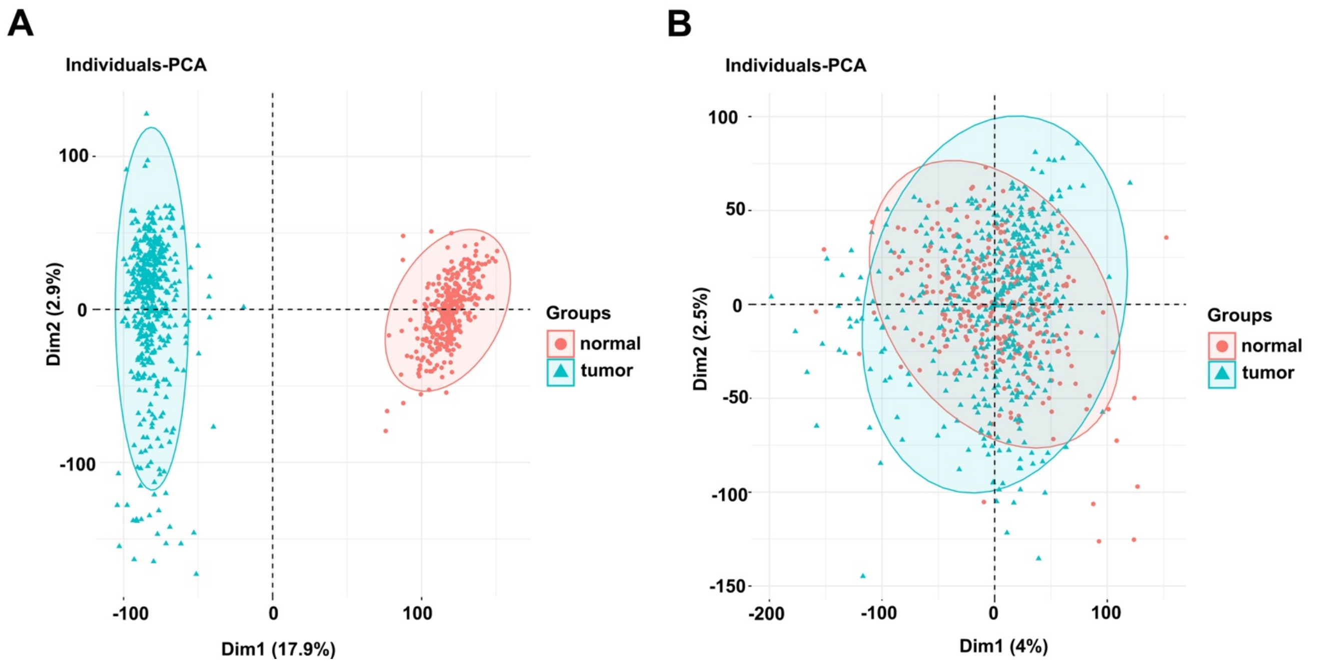

FIGURE S1 Results of normalization by principal component analysis for TCGA-SKCM dataset.

Two boxplots of normal and tumor groups performed and visualized before (A) and after (B) batch effect removement.

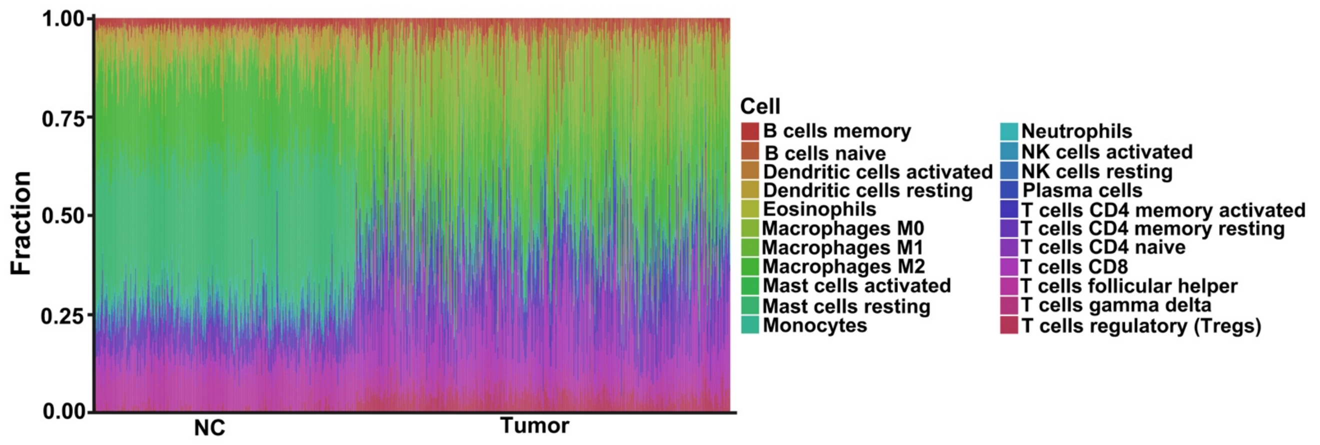

FIGURE S2 Difference analysis of immune cells between melanoma and healthy tissues using the CIBERSORTx algorithm. Barplots of different fractions of 22 immune cells between melanoma patients and healthy tissues. NC represents healthy control, T represents Tumors.

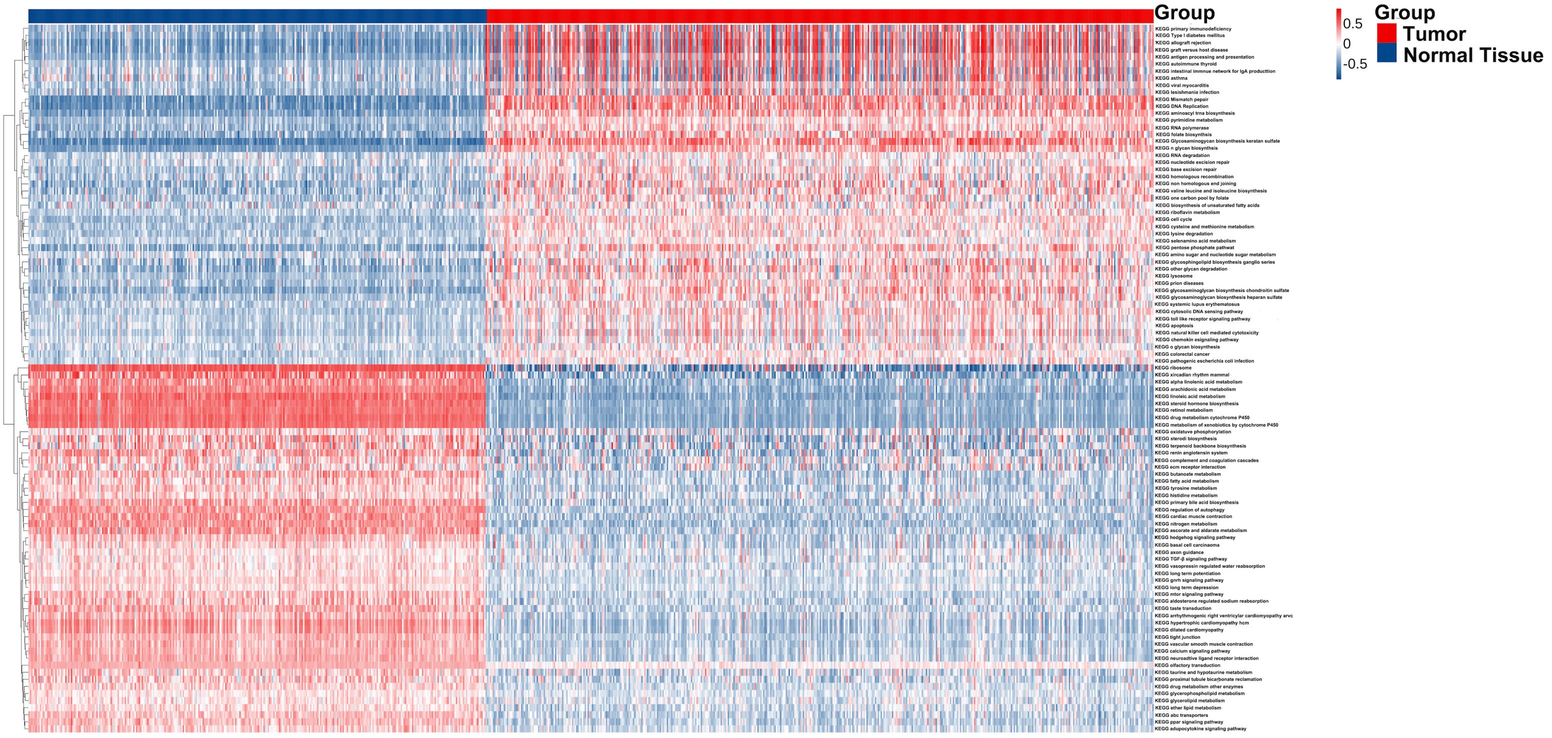

Group
KEGG primary immunodeficiency
KEGG Type I diabetes mellitus
KEGG allograft rejection
KEGG graft versus host disease
KEGG antigen processing and presentation
KEGG autoimmune thyroid
KEGG intestinal immnue network for IgA producttion
KEGG asthma
KEGG viral myocarditis
KEGG lesishmania infection
KEGG Mismatch pepair
KEGG DNA Replication
KEGG aminoacyl trna biosynthesis
KEGG pyrimidine metabolism
KEGG RNA polymerase
KEGG folate biosynthsis
KEGG Glycosaminogycan biosynthesis keratan sulfate
KEGG n glycan biosynthsis
KEGG RNA degradation
KEGG nucleotide excision repair
KEGG base excision repair
KEGG homologous recombination
KEGG non homologous end joining
KEGG valine leucine and isoleucine biosynthesis
KEGG one carbon pool by folate
KEGG biosynthesis of unsaturated fatty acids
KEGG riboflavin metabolism
KEGG cell cycle
KEGG cysteine and methionine metabolism
KEGG lysine degradation
KEGG selenamino acid metabolism
KEGG pentose phosphate pathwat
KEGG amino sugar and nucleotide sugar metabolism
KEGG glycosphingolipid biosynthesis ganglio series
KEGG other glycan degradation
KEGG lysosome
KEGG prion diseases
KEGG glycosaminoglycan biosynthesis chondroitin sulfate
KEGG glycosaminoglycan biosynthesis heparan sulfate
KEGG systemic lupus erythematosus
KEGG cytosolic DNA sensing pathway
KEGG toll like receptor signaling pathway
KEGG apoptosis
KEGG natural killer cell mediated cytotoxicity
KEGG chemokin esignaling pathway
KEGG o glycan biosynthesis
KEGG colorectal cancer
KEGG pathogenic escherichia coli infection
KEGG ribosome
KEGG xircadian rhythm mammal
KEGG alpha linolenic acid metabolism
KEGG arachidonic acid metabolism
KEGG linoleic acid metabolism
KEGG steroid hormone biosynthesis
KEGG retinol metabolism
KEGG drug metabolism cytochrome P450
KEGG metabolism of xenobiotics by cytochrome P450
KEGG oxidatuve phosphorylation
KEGG sterodi biosynthesis
KEGG terpenoid backbone biosynthesis
KEGG renin angiotensin system
KEGG complement and coagulation cascades
KEGG ecm receptor interaction
KEGG butanoate metabolism
KEGG fatty acid metabolism
KEGG tyrosine metabolism
KEGG histidine metabolism
KEGG primary bile acid biosynthesis
KEGG regulation of autophagy
KEGG cardiac muscle contraction
KEGG nitrogen metabolism
KEGG ascorate and aldarate metabolism
KEGG hedgehog signaling pathway
KEGG basal cell carcinaoma
KEGG axon guidance
KEGG TGF-β signaling pathway
KEGG vasopressin regulated water reabsorption
KEGG long term potentiation
KEGG gnrh signaling pathway
KEGG long term depression
KEGG mtor signaling pathway
KEGG aldosterone regulated sodium reabsorption
KEGG taste transduction
KEGG arrhythmogenic right ventricylar cardiomyopathy arvc
KEGG hypertrophic cardiomyopathy hcm
KEGG dilated cardiomyopathy
KEGG tight junction
KEGG vascular smooth muscle contraction
KEGG calcium signaling pathway
KEGG neuroadtive ligand receptor interaction
KEGG olfactory transduction
KEGG taurine and hypotaurine metabolism
KEGG proximal tubule bicarbonate reclamation
KEGG drug metabolism other enzymes
KEGG glycerophospholipid metabolism
KEGG glycerolipid metabolism
KEGG ether lipid metabolism
KEGG abc transporters
KEGG ppar signaling pathway
KEGG adupocytokine signaling pathway
0.5
0
-0.5
Group
Tumor
Normal Tissue

FIGURE S3 Heatmap of KEGG pathways identified by GSVA analysis in an individual sample. The blue boxplot represents healthy control; the red boxplot represents Tumors.
