# Supplementary material for: Correlation of tumor‐infiltrating immune cells of melanoma with overall survival by immunogenomic analysis
Source: Cancer Med. 2020 Sep 15;9(22):8444–56. doi: 10.1002/cam4.3466 (PMC7666744; doi:10.1002/cam4.3466)
Supplement: Supplementary file 2 — Table S1 [file CAM4-9-8444-s002.docx]

**TABLE S1** KEGG pathways for genes in melanoma

| KEGG pathways | Core enrichment Gene |
| --- | --- |
| Intestinal immune network for IgA | *HLA-DRB4 TNFRSF13C HLA-DRB5*  *HLA-DOA HLA-DOB HLA-DQA2*  *HLA-DRB3 CD80 TNFSF13B CCL27*  *HLA-DRB1 CD86 CD28 IL6 TNFSF13*  *IL15RA IL15 ICOSLG ITGA4 CCR9 IL10*  *TGFB1 ICOS MAP3K14 HLA-DMB*  *CXCR4 CCL25 TNFRSF17 HLA-DRA*  *HLA-DMA CCL28 LTBR PIGR*  *IL2 CD40LG HLA-DPA1 CD40 IL4*  *AICDA HLA-DPB1 HLA-DQA1 IL5*  *HLA-DQA2 HLA-DQB1 TNFRSF13B*  *ITGB7 CXCL12 CCR10 MADCAM1* |
| Primary immunodeficiency | *ZAP70 CD4 TNFRSF13C IKBKG IL2RG*  *RFXAP TAP2 RFX5 CD8A CD8B*  *TAP1 ICOS UNG IL7R ADA*  *CD40LG CD40 AICDA BTK DCLRE1C*  *BLNK CD19 RAG2 RAG1 CD3D*  *CD3E RFXANK AIRE CD79A PTPRC TNFRSF13B JAK3 IGLL1 CIITA LCK* |
| TGF-β signaling pathway | *TFDP1 NOG TNF GDF7 INHBB*  *INHBC COMP INHBA THBS4*  *RHOA CREBBP ROCK1 ID1 ID2*  *RPS6KB1 RPS6KB2 THBS1 CUL1*  *LOC728622 ID4 SMAD3 MAPK3 RBL2*  *SMAD4 RBL1 NODAL SMAD1 MYC*  *SMAD2 MAPK1 SMURF2 SMURF1 EP300*  *BMP8A GDF5 SKP1 CHRD DCN*  *TGFB2 TGFB1 IFNG CDKN2B*  *PPP2CB PPP2CA PPP2R1A ID3*  *SMAD5 RBX1 FST PITX2 PPP2R1B*  *TGFBR2 AMHR2 LTBP1 LEFTY1 AMH*  *TGFBR1 SMAD9 LEFTY2 SMAD7*  *ROCK2 TGFB3 SMAD6 BMPR2 GDF6*  *BMPR1A BMPR1B ACVRL1 ACVR2B*  *ACVR2A ACVR1 BMP4 E2F5*  *BMP2 ACVR1C E2F4 SP1 BMP7*  *BMP8B ZFYVE9 BMP5 BMP6*  *ZFYVE16 THBS3 INHBE THBS2* |
